# Reduced Triacylglycerol Mobilization during Seed Germination and Early Seedling Growth in Arabidopsis Containing Nutritionally Important Polyunsaturated Fatty Acids

**DOI:** 10.3389/fpls.2016.01402

**Published:** 2016-09-26

**Authors:** Pushkar Shrestha, Damien L. Callahan, Surinder P. Singh, James R. Petrie, Xue-Rong Zhou

**Affiliations:** ^1^CSIRO Agriculture and FoodCanberra, ACT, Australia; ^2^Metabolomics Australia, School of Biosciences, University of MelbourneMelbourne, VIC, Australia; ^3^Centre for Chemistry and Biotechnology, School of Life and Environmental Science, Deakin UniversityMelbourne, VIC, Australia

**Keywords:** triacylglycerol utilization, lipidomics, ω3 LC-PUFA, SDA, ARA, DHA, Arabidopsis, seed germination

## Abstract

There are now several examples of plant species engineered to synthesize and accumulate nutritionally important polyunsaturated fatty acids in their seed triacylglycerols (TAG). The utilization of TAG in germinating seeds of such transgenic plants was unknown. In this study, we examined the TAG utilization efficiency during seed germination in transgenic Arabidopsis seeds containing several examples of these fatty acids. Seed TAG species with native fatty acids had higher utilization rate than the TAG species containing transgenically produced polyunsaturated fatty acids. Conversely, quantification of the fatty acid components remaining in the total TAG after early stages of seed germination revealed that the undigested TAGs tended to contain elevated levels of the engineered polyunsaturated fatty acids (PUFA). LC-MS analysis further revealed asymmetrical mobilization rates for the individual TAG species. TAGs which contained multiple PUFA fatty acids were mobilized slower than the species containing single PUFA. The mobilized engineered fatty acids were used in *de novo* membrane lipid synthesis during seedling development.

## Introduction

Plants synthesize fatty acids and store them as triacylglycerols (TAG) in seeds which are subsequently utilized during seed germination and early seedling development for energy before seedlings are fully established and photosynthetically sustainable. Mobilization of TAG is initiated by the activity of seedling-specific TAG lipases which liberate fatty acids from TAG molecules. The free fatty acids can then be converted to acetyl-CoA units, which are the precursors of sugar synthesis, via β-oxidation cycle in the peroxisome.

More than 200 unique fatty acids have been identified in plant species. These are classified according to chain length, number of carbon atoms, type and arrangement of unsaturated bonds, and presence of functional groups (Badami and Patil, 1980). This huge structural diversity means that seed oils often consist of a wide range of TAG species with different fatty acid combinations. Seed TAG lipases often have selectivity for common fatty acid combinations in TAG found in that species. For instance, Lin et al. (1986) showed high activity of canola seed lipase toward triolein, trilinolein, and trierucin, fatty acids which naturally dominate the *Brassica napus* oil profile. Similarly, corn seed lipase was found to have high activity toward tricaprin and trilinolein and the lupin seed lipase was more active on TAGs containing saturated fatty acids than containing unsaturated fatty acids (Huang et al., 1988; Sanz and Olias, 1990). Sunflower seed lipase have shown preference for triacylglycerols with mono-unsaturated fatty acids (Sagiroglu and Arabaci, 2005). Bean lipases showed specificity for short and medium chain fatty acids (Enujiugha et al., 2004). In addition, TAG lipases from wild plant species have been demonstrated to have higher specificity for the dominant fatty acids in their seeds (Lin et al., 1986; Hellyer et al., 1999). For example, castor bean seeds contain 90% ricinoleic acid, *Vernonia galamensis* seeds contain 80% vernolic acid and *Cuphea sps.* seeds contain >80% capric acid, their TAG lipases have highest specificities for triricinolein (Lin et al., 1986), trivernolin (Ncube et al., 1995), and tricaprin (Hellyer et al., 1999), respectively, compared to TAGs containing other fatty acids.

Recently, various new fatty acids have been introduced to seed oil in crop species by genetic engineering (Ruiz-López et al., 2012; Vanhercke et al., 2013). In particular, the production of pharmaceutically and nutraceutically important polyunsaturated fatty acids (PUFA) has been reported in seeds of various species including 70% (v/v) gamma-linolenic acid (C18:3^Δ6, 9, 12^, GLA) in safflower seed (Nykiforuk et al., 2012), 13% stearidonic acid (SDA) in linseed (Ruiz-López et al., 2009), 10% arachidonic acid (ARA) in canola seed (Petrie et al., 2012a), 25% eicosapentaenoic acid (EPA) in *Brassica carinata* seed (Cheng et al., 2010), 15% docosahexaenoic acid (DHA) in Arabidopsis seed (Petrie et al., 2012b), and 12% DHA in Camelina seed (Petrie et al., 2014). Unusual fatty acids such as vernolic acid (Zhou et al., 2006), ricinoleic acid (Kim et al., 2011), and capric acid (Knutzon et al., 1999) have also been produced in various species using appropriate transgenes.

Such drastic alteration of fatty acid composition of seed TAGs may affect TAG lipolysis during germination and thus affect the seed vigor (Lin et al., 1986; Liu et al., 2002). Wang et al. (2001) reported a significant negative correlation between elevated saturate lipids and germination under some conditions in soybean seeds, suggesting that increased stearate levels may be more detrimental to the viability and vigor of seeds than increased palmitate. However, there is not enough knowledge about the specificities of endogenous seed lipases toward engineered fatty acids accumulated in TAG and their effect on early seedling development to predict similar responses toward polyunsaturated fatty acids.

Here we describe an investigation of the utilization of TAG during seed germination in transgenic Arabidopsis seeds that have been engineered to produce PUFA. We compare the rate of utilization of three engineered fatty acids, namely SDA (18:4^Δ6, 9, 12, 15^), ARA (20:4^Δ5, 8, 11, 14^) and DHA (22:6^Δ4, 7, 10, 13, 16, 19^), to the native fatty acids in TAG. It was found that the utilization of the engineered fatty acids was slower than native fatty acids from TAG, leading to the elevated levels of engineered PUFA in the remaining TAGs. The individual TAG species that contained more PUFA molecules showed slower mobilization rate than TAG species containing fewer PUFA molecules. We also showed that the engineered fatty acids were used in *de novo* membrane lipid synthesis during seedling development.

## Materials and methods

### Plant materials

Transgenic seeds containing various PUFA were generated in our lab. SDA-containing line (IK14) expressed the *Micromonas pusilla* Δ6-desaturase to produce SDA (Petrie et al., 2010). The ARA-containing line (FW10) expressed the *Isochrysis galbana* Δ9-elongase, the *Pavlova salina* Δ8-, and Δ5-desaturases (Petrie et al., 2012a) in *fad3/fae1* double mutant MC49 (Zhou et al., 2006). The DHA-containing line expressed the *Lachancea kluyveri* Δ12-desaturase, *Pichia pastoris* ω3-desaturase, *M. pusilla* Δ6-desaturase, *Pyramimonas cordata* Δ6- and Δ5-elongases and *P. salina* Δ5- and Δ4-desaturases (Petrie et al., 2012b). Transgenic homozygous lines and wild type control Columbia plants were grown alongside each other in a glasshouse to maintain uniformity of seeds. The lines were designated as SDA-, ARA-, and DHA-lines below.

### Seedling growth

After harvest, seeds were cleaned and desiccated overnight in a desiccator. Seeds were weighed into batches of approximately 10 mg for each sample. Triplicate samples from Columbia, SDA-, ARA-, and DHA-line were sown for each time point on a single layer of wet filter paper in a 12 cm plastic petri dish. Each petri dish contained 15 samples of an individual line. Seeds were imbibed for 4 days at 4°C in the dark and then grown under uniform light (150 μmol/m^2^/s) and under light/dark cycle (8/16 h) at 24°C. Germination of seeds were recorded everyday between 0 and 4 days after imbibition (DAI). Triplicate seed and seedling samples were collected at 24 h increments until 4 DAI in 2 mL Eppendorf tubes. Samples were then frozen in liquid nitrogen and stored at −80°C until total lipid was extracted.

### Lipid extraction and analysis

Seed and seedling tissue were homogenized in an Eppendorf tube with a metal ball in a Reicht Tissue Lyser (Qiagen). Lipids were then extracted from the homogenized tissues as previously described (Zhou et al., 2013) with the exception that ice-cold solutions were used and the extractions performed on ice. Solvent was then evaporated from the lipid samples on a 40°C ceramic plate under nitrogen flow before resuspension of the lipids in known volumes of CHCl_3_ per mg seed dry weight. Samples were stored at −20°C.

Neutral lipid classes were fractionated by loading total lipid on a thin layer chromatography (TLC) silica plate (Silica gel 60, MERCK) with a solvent mixture of hexane:diethyl ether:acetic acid (70:30:1, v/v). Polar lipid classes were separated using chloroform:methanol:acetic acid:water (90:15:10:3, v/v). The TLC tank was pre-filled with argon to reduce PUFA oxidation. Lipid spots and bands were identified by running authentic TLC standards (NuChek PREP, Inc. USA) alongside the samples. These were visualized under UV light after spraying the plate with 0.001% primuline, marking outlines with a pencil, lightly spraying with water, and collecting into argon-filled glass vials. Methanol (0.1 mL) was added to the vials and evaporated under N_2_ to remove moisture from the lipid/silica samples.

Fatty acid profiles and amounts of fatty acids present in the lipid classes were determined by incubating silica spots in 1N methanolic HCl (Supelco, Bellefonte, PA) together with known amounts of Tri-17:0-TAG (NuChek Prep) as an internal standard and running fatty acid methyl esters thus produced in GC as previously described (Zhou et al., 2011), and calculated based on the weight of starting seeds.

### LC-MS analysis

Lipids from 1 mg seed extracts were dissolved 1 mL of butanol:methanol (1:1, v/v) containing 10 mM butylated hydroxytoluene. Individual TAG were analyzed using LC-MS as previously described (Zhou et al., 2014). Selected neutral lipids (TAG and DAG), phosphatidylcholine (PC) and galactolipids (monogalactosyldiacylglycerol MGDG; digalactosyldiacylglycerol, DGDG; sulfoquinovosyl diacylglycerol, SQDG) were analyzed using multiple reaction monitoring (MRM) mode. Neutral lipids were targeted to those containing combinations of the following major fatty acids: 16:0, 18:0, 18:1, 18:2, 18:3, 18:4, 20:0, 20:1, 20:2, 20:3, 20:4, 20:5, 22:4, 22:5, 22:6, while phospholipids were targeted containing 16:0, 16:3, 18:0, 18:1, 18:2, 18:3, 18:4, 20:0, 20:1, 20:2, 20:3, 20:4, 20:5, 22:5, 22:6. Parent MRM masses for each TAG were based on ammonium adducts. The product ions for MRM transitions were based on the neutral loss of 18:3, 18:4, 20:4 or 22:6 fatty acids. This enabled the measurement of ALA (18:3)-, SDA (18:4)-, ARA (20:4)-, or DHA (22:6)- containing TAGs. Semi-quantitation was carried out on TAG and DAG with a single point external calibration of a 50 μM tristearin and distearin mixed standard. Phosphatidylcholine was quantified with 10 μM of di-18:0-PC external standard, and MGDG, DGDG, SQDG were quantified with 10 μM of di-18:3-MGDG, di-18:3-DGDG, 16:0/18:3-SQDG external standards.

## Results

### Fatty acid profile and oil content in transgenic seeds

Total fatty acid profiles of seeds from these lines showed the presence of newly engineered PUFA products (Table 1). In the seeds of SDA-line, the engineered fatty acid products GLA and SDA constituted 4.6 and 8.6% of total fatty acids, respectively. ARA-line seeds produced 15.7% ARA and 1% dihomogamma linolenic acid (DGLA), while DHA-line seeds contained 5.3% SDA, 1.3% eicosatetraenoic acid (ETA), 2.0% EPA and 11.6% DHA, together with other minor engineered fatty acids and normal fatty acids. Lower levels of oil content were observed in transgenic seeds compared to Columbia control. Columbia seeds contained 28.6% oil by dry weight and seeds of SDA, ARA, and DHA-lines contained 24.7, 22.0, and 18.9% oil by dry weight in their seeds, respectively.

**Table 1:** **Oil content and fatty acid profiles in wild type and transgenic lines of Arabidopsis seeds**.

**Fatty acid**		**Columbia**	**SDA line**	**ARA line**	**DHA line**
	16:0	7.6 ± 0.0	7.2 ± 0.0	8.3 ± 0.0	10.4 ± 0.4
	18:0	3.0 ± 0.0	3.8 ± 0.0	3.8 ± 0.0	3.6 ± 0.1
	18:1ω7	1.5 ± 0.0	2.5 ± 0.0	2.8 ± 0.0	3.9 ± 0.2
	20:0	2.1 ± 0.0	2.2 ± 0.0	0.7 ± 0.0	1.6 ± 0.1
	20:1ω9/11	20.0 ± 0.1	14.4 ± 0.1	1.9 ± 0.0	11.2 ± 0.4
	22:1	1.7 ± 0.0	1.1 ± 0.0	tr	0.5 ± 0.0
	Minor	1.3 ± 0.0	1.4 ± 0.0	1.1 ± 0.0	1.5 ± 0.1
	18:1	14.2 ± 0.1	16.1 ± 0.1	25.0 ± 0.3	7.1 ± 0.4
	18:2	28.6 ± 0.1	23.8 ± 0.1	31.6 ± 0.2	6.0 ± 0.2
	18:3	17.6 ± 0.0	12.3 ± 0.0	0.5 ± 0.0	30.8 ± 0.5
Omega-6	18:3ω6	−	**4.6** ± 0.0	tr	0.5 ± 0.0
	20:2ω6	1.8 ± 0.0	1.2 ± 0.0	6.0 ± 0.2	0.6 ± 0.0
	20:3ω6	−	0.1 ± 0.0	1.0 ± 0.0	0.1 ± 0.0
	20:4ω6	−	−	**15.7** ± 0.5	−
	22:4ω6	−	−	0.4 ± 0.0	−
	22:5ω6	−	−	tr	−
Omega-3	18:4ω3	−	**8.6** ± 0.1	−	**5.3** ± 0.2
	20:3ω3	0.4 ± 0.0	0.5 ± 0.0	0.4 ± 0.0	1.0 ± 0.0
	22:3ω3	0.1 ± 0.0	tr	0.1 ± 0.0	0.1 ± 0.0
	20:4ω3	−	0.1 ± 0.0	tr	**1.3** ± 0.0
	20:5ω3	−	−	0.5 ± 0.0	**2.0** ± 0.1
	22:5ω3	−	−	−	0.9 ± 0.0
	22:6ω3	−	−	−	**11.6** ± 0.2
	% oil by seed wt.	28.6	24.7	22.0	18.9

The major engineered fatty acids products are shown in bold. The errors denote standard deviation.

### Changes in fatty acid profiles of TAG in seedlings

**Figure 1:**
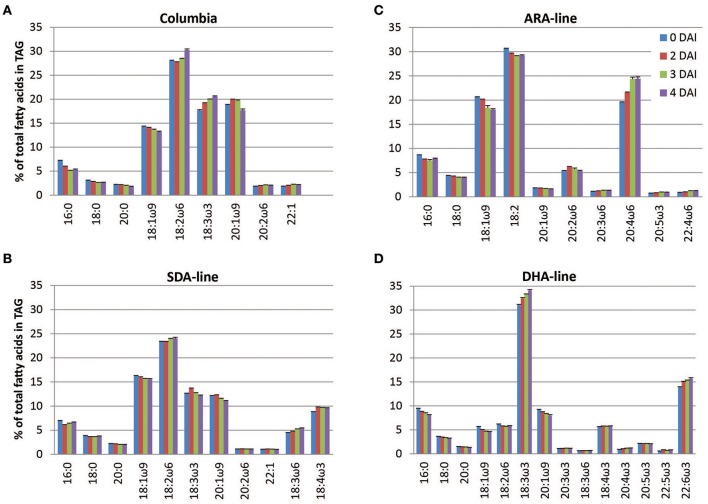
**Profiles of major and engineered fatty acids of triacylglycerols in seedlings of Arabidopsis lines**. Panels **(A–D)** are for Columbia, SDA-, ARA-, and DHA-lines, respectively. The error bars denote standard deviation from triplicates. ALA in the ARA-line was minor component, and not included here.

The fatty acid profiles of TAG changed during seedling development in both Columbia and the transgenic lines. In Columbia, proportions of saturated and monounsaturated fatty acids in TAGs decreased while there were some increases in the proportions of polyunsaturated fatty acids (Figure 1A). This suggested that the utilization of saturated and monounsaturated fatty acids was faster than that of polyunsaturated fatty acids. In the SDA-line, proportions of GLA and SDA increased by 0.9% and 0.8% of total fatty acid in TAG, respectively, at 4 DAI (Figure 1B). A higher proportion of PUFA was observed in the ARA-line, where ARA increased by 4.8% at 4 DAI (Figure 1C). The proportions of minor component 20:2ω6 and DGLA were also increased (data not shown). ALA was a minor component in the ARA-line owing to a *fad3* knockout in this line (Table 1). The DHA-line showed an increase in the proportions of DHA and ALA by 1.8 and 3.1%, respectively, at 4 DAI (Figure 1D). The proportions of SDA and ETA were also increased (data not shown). These results all suggested a slower rate of utilization of engineered polyunsaturated fatty acid containing TAGs.

**Figure 2:**
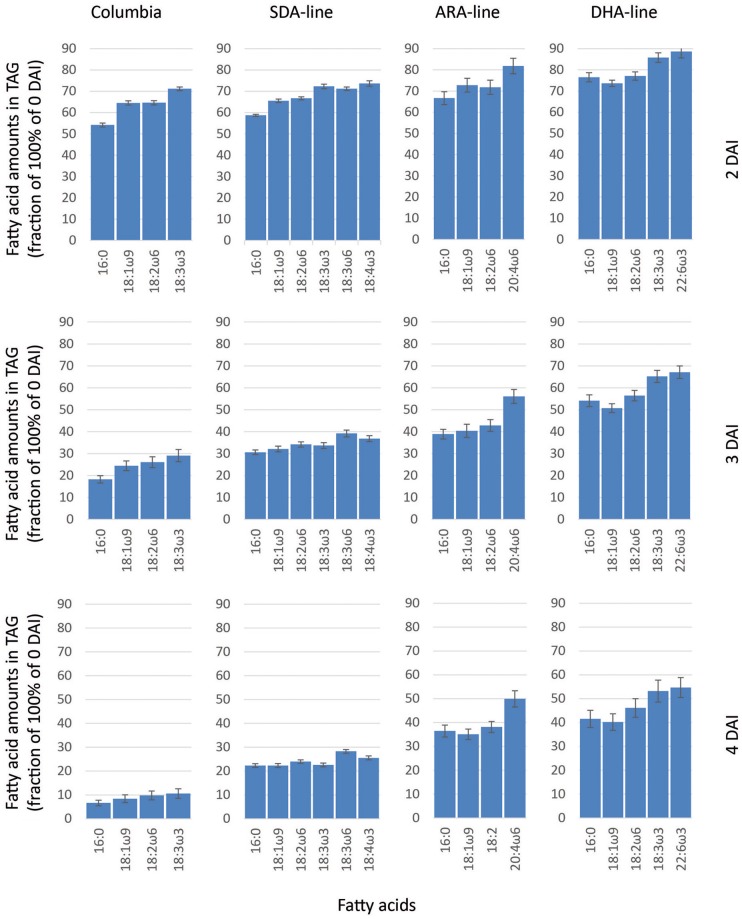
**Relative fatty acid amounts in remaining TAG (compared to 0 DAI) after 2, 3, and 4 DAI in Columbia, SDA-, ARA-, and DHA-lines of Arabidopsis**. The error bars denote standard deviation from triplicates.

Quantification of individual fatty acids in TAGs during seedling development relative to 0 DAI showed an asymmetrical pattern of mobilization of individual fatty acids from TAG. In Columbia, longer chain and polyunsaturated fatty acids had higher remaining amounts compared to shorter chain and saturated fatty acids, indicating that longer chain and polyunsaturated fatty acids were mobilized at a slower rate from TAGs (Figure 2). At 2 DAI, 54% of the shorter chain and saturated palmitic acid remained in TAG compared to 71% of polyunsaturated ALA remained, suggesting 46% of palmitic acid and 29% of ALA were mobilized. A similar pattern was observed in Columbia at 3 DAI and 4 DAI when the remaining TAGs were further decreased. In the SDA-line, 69% of palmitic acid and 66% of ALA were liberated from TAG at 3 DAI, compared to 61 and 63% of engineered fatty acids, GLA and SDA (Figure 2). In the ARA-line, 61, 60, and 57% of native palmitic, oleic and linoleic acids were utilized from TAG at 3 DAI (Figure 2). In contrast, only 47, 44, 44, and 40% of engineered PUFAs, namely DGLA, ARA, EPA, and 22:4n6 were utilized from TAG (Figure 2 and data not shown). Similarly, in the DHA-line 46, 49, 44, and 35% of native palmitic acid, OA, LA, and ALA were mobilized from TAG at 3 DAI, while only 38, 21, 40, 25, and 33% of engineered PUFAs, namely SDA, ETA, EPA, DPA, and DHA were utilized from TAG, respectively (Figure 2 and data not shown). Such asymmetrical pattern of TAG mobilization could be observed in all lines with engineered fatty acids during the germination from 2 to 4 DAI. The presence of engineered fatty acids with longer chain and higher desaturation levels also tended to affect the mobilization of the native fatty acids, for example, comparing the remaining palmitic acid or ALA between the four lines at the same DAI (Figure 2).

### Reduced TAG mobilization during seedling development

**Figure 3:**
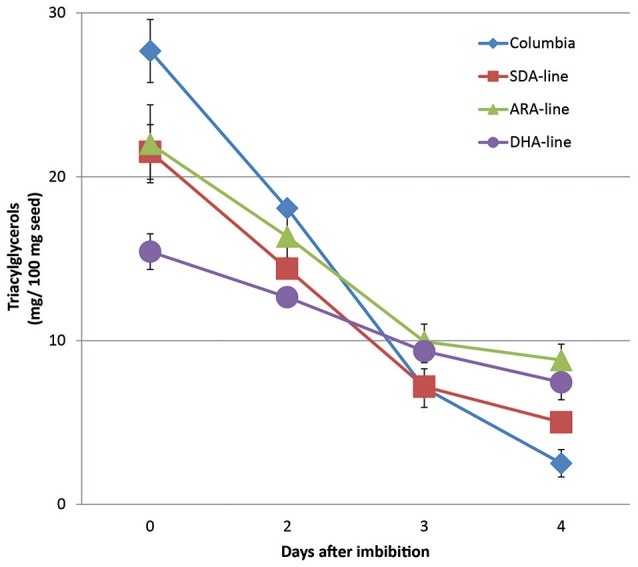
**TAG amount remained in seedlings per 100 mg original seeds after imbibition**. The error bars denote standard deviation from triplicates.

In addition to reduced PUFA liberation compared to other fatty acids, the total amounts of mobilized TAG were also reduced during seedling development. The remaining TAG compared to initial TAGs (0 DAI) was used to indicate the TAG mobilization. As shown in Figure 3, the TAG utilization was slower in all transgenic lines compared to Columbia. In Columbia, TAG amount dropped from 28 mg per 100 mg seeds used at 0 DAI to 4 mg at 4 DAI, indicating 86% of seed TAG was mobilized. In contrast, SDA, ARA, and DHA-lines showed mobilization of 78, 60, and 50% of TAG, respectively. It should be pointed out that ARA-line was in MC49 (*fad3/fae1* double mutant) background, instead of Columbia, to minimize the competition of ω3 pathway on Δ6-desaturase for maximal level of ω6 product ARA. Our initial results showed reduced total TAG utilization in ARA-line at 4 DAI compared to MC49 parent line (data not shown). The MC49 was not included in the following experiments with detailed TAG species analysis from 0 to 4 DAI by LC-MS, especially there was very low levels of ALA due to the mutation.

### Seedling development

Some delay of seed germination and seedling development of transgenic plants was observed under normal growth conditions (data not shown). Here we further tested the germination of T_3_ homozygous transgenic seeds on filter paper. As shown in Figure S1, Columbia seeds germinated and developed vigorously during the experimental period of 4 DAI, while the transgenic seeds showed notable differences in development pattern. SDA-line seeds showed no significant delay in germination or seedling development. Seed germination and seedling development were delayed in the ARA-line and DHA-lines. Once the seedlings were established, however, no obvious phenotypical differences between transgenic lines and wild type plants were apparent.

### Mobilization of TAG and DAG molecular species

Differences in the mobilization of different fatty acids in TAG shown above could be due to certain TAG molecular species being more susceptible to seed lipase. Thus, the individual seedling TAG and DAG species were analyzed by LC-MS and quantified on the basis of an external standards. Arabidopsis seeds contained a large number of TAG species, so only TAG species containing ALA or the dominant engineered PUFAs SDA, ARA, and DHA were analyzed. TAG containing a single PUFA was targeted with the remaining two fatty acids being identified on the basis of total number of carbon atoms and double bonds in the TAG molecule. Due to relatively simpler profiles, all species were included in DAG, PC, and galactolipid analysis.

**Figure 4:**
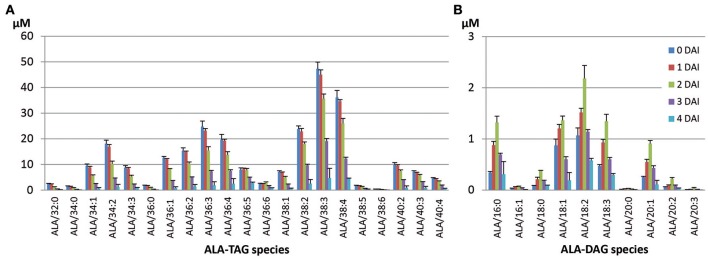
**Mobilization of ALA-containing TAG (A) and accumulated DAG (B) species during seedling development in Columbia**. The two numbers in TAG species denote total number of carbons and total number of double bonds of two fatty acids in the TAG species.

In Columbia, the major ALA-containing TAG species were ALA/38:3 (17.8%) and ALA/38:4 (13.6%) at 0 DAI, which potentially contained ALA with 18:2/20:1, or with 18:3/20:1 as major species, respectively (Table 2). Among all ALA-containing TAG species, species containing saturated fatty acid for other two acyl chains, especially both were saturated, were mostly mobilized at 4 DAI, while three- or two-ALA species were utilized at lower levels (Figure 4A, Table 2). Interestingly, most unused ALA-TAG at 4 DAI was tri-ALA TAG. Also, among mono- or di-ALA-TAG species, 18:2-containing species mobilized at lower degree compared to the species that contained saturates or monounsaturates (MUFA).

**Table 2:** **Mobilization of ALA-containing TAG species on 4 DAI during seedling development in Columbia**.

^*^ALA-TAG	**Molecular species**			**Mol% (0 DAI)**	**Relative amount (% of 0 DAI)**
ALA/36:0	ALA/16:0/20:0	ALA/18:0/18:0		0.7 ± 0.0	6.3 ± 0.1
ALA/32:0	ALA/16:0/16:0			0.9 ± 0.0	6.4 ± 1.0
ALA/34:0	ALA/16:0/18:0			0.6 ± 0.0	7.0 ± 0.4
ALA/36:1	ALA/16:0/20:1	ALA/18:0/18:1		4.7 ± 0.1	8.8 ± 0.5
ALA/34:1	ALA/16:0/18:1			3.6 ± 0.0	9.1 ± 0.4
ALA/38:1	ALA/16:0/22:1	ALA/18:0/20:1	ALA/18:1/20:0	2.7 ± 0.0	9.8 ± 0.7
ALA/34:2	ALA/16:0/18:2			6.8 ± 0.0	10.1 ± 0.6
ALA/36:3	ALA/18:0/18:3	ALA/18:1/18:2		9.3 ± 0.1	10.9 ± 0.5
ALA/34:3	ALA/16:0/18:3			3.3 ± 0.1	11.5 ± 1.1
ALA/36:2	ALA/16:0/20:2	ALA/18:0/18:2	ALA/18:1/18:1	5.8 ± 0.0	11.9 ± 0.3
ALA/38:4	ALA/18:2/20:2	ALA/18:3/20:1		13.6 ± 0.1	12.4 ± 0.9
ALA/40:2	ALA/18:1/22:1	ALA/20:0/20:2	ALA/20:1/20:1	3.8 ± 0.0	13.7 ± 0.1
ALA/38:2	ALA/18:0/20:2	ALA/18:1/20:1	ALA/18:2/20:0	9.0 ± 0.2	14.6 ± 0.8
ALA/38:3	ALA/18:1/20:2	ALA/18:2/20:1	ALA/18:3/20:0	17.8 ± 0.3	14.6 ± 1.2
ALA/40:4	ALA/18:3/22:1	ALA/20:2/20:2		1.7 ± 0.0	15.5 ± 0.7
ALA/40:3	ALA/18:2/22:1	ALA/20:1/20:2		2.7 ± 0.1	16.6 ± 0.5
ALA/36:4	ALA/18:1/18:3	ALA/18:2/18:2		7.4 ± 0.2	18.5 ± 1.6
ALA/36:5	ALA/18:2/18:3			3.0 ± 0.1	36.3 ± 5.5
ALA/36:6	ALA/18:3/18:3			0.9 ± 0.0	39.4 ± 0.9

* Sum of carbon and double bond numbers in other two acyls of ALA-TAG. Species containing minor fatty acids (< 0.5%) are not shown.

There was a trend toward higher mobilization of TAG species that contained shorter chain, saturated (or not highly unsaturated) fatty acids. Conversely, TAG species containing longer chains and higher levels of unsaturation were mobilized at slower rates. All ALA-DAG species in Columbia were mobilized swiftly during 4 DAI period of seedling development (Figure 4B). The dominant ALA-containing TAG species were ALA/38:3 and ALA/38:4, while the dominant ALA-containing DAG species were ALA/18:2 and ALA/18:3, likely from TAG containing ALA/18:2/20:1 and ALA/18:3/20:1, considering the abundance of 20:1 in Columbia TAG.

**Figure 5:**
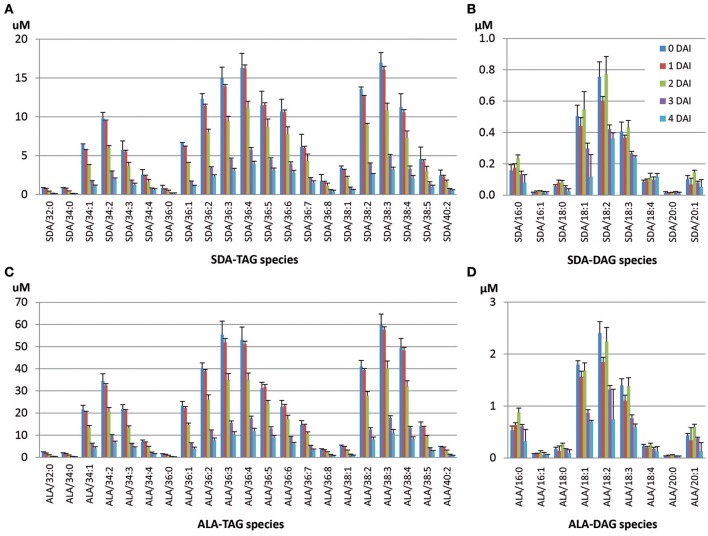
**Mobilization of SDA- or ALA-containing TAG and accumulated DAG species during seedling development in the SDA-line**. **(A)** SDA-containing TAG species, **(B)** SDA-containing DAG species, **(C)** ALA-containing TAG species, **(D)** ALA-containing DAG species. The two numbers in species denotes total number of carbons and total number of double bonds of two or single fatty acids in the TAG or DAG species, respectively.

In the SDA-line, ALA-containing or SDA-containing TAG and DAG species were targeted for LC-MS analysis. Detailed mobilization analysis of SDA-containing TAG species is shown in Figure 5, and the remaining TAG based on the estimated number of SDA in TAG at 4 DAI relative to 0 DAI is shown in Table 3. SDA/38:3 (mono-SDA TAG, 10.6%) and SDA/36:4 TAG (mixture of mono- and di-SDA species, 10.2%) made up the highest proportions of total SDA-containing TAG species. Tri-SDA TAG constituted only 1.1%. Di- and tri-SDA TAGs were mobilized in lower amounts compared to mono-SDA TAGs. At 4 DAI, mono-SDA TAG species were remained at the levels of 14–20% of the original level. However, remaining di- and tri-SDA TAGs were at the levels of 20–27%, indicating less mobilization of those species (Table 3). The distribution of these species during seedling development is shown in Figure 5A, with the analyzed TAG species all reduced at different levels during the 4 DAI period. Major SDA-containing TAG species were SDA/36:3, SDA/36:4, SDA/38:3. The seedlings also showed slightly increased accumulation of di-SDA DAG species while the major mono-SDA containing DAG reduced (Figure 5B). This relative enrichment was possibly due to the preferential lipolysis of normal fatty acid from di-SDA TAG species and/or due to lower specificity of DAG lipase to SDA. It is worth to mention that the DAG accumulation during the seedling development was the combination of DAGs generated from TAG mobilization and the endogenous seed DAGs which were also being mobilized. The TAG mobilization of 1–2 DAI or 2–3 DAI was higher than that of 0–1 DAI, generating more DAG. This could be the reason for transient increase of some DAG species at 2 DAI than 1 DAI (Figure 5B). Analysis of ALA-containing TAG species in the SDA-line seedlings showed that the ALA-containing species that also contained SDA were less mobilized than species without SDA (Figure 5C, Table 3). Similarly, ALA/18:4-DAG species mobilized at a slower rate, compared to other ALA-containing DAG species without SDA, as showed in Figure 5D that ALA/18:4 remained similar amount during the time course, while other DAG species decreased significantly from 0 to 4 DAI.

**Table 3:** **Mobilization of SDA- and ALA-containing TAG species on 4 DAI during seedling developments in SDA-line**.

^*^Species	**SDA- or ALA** + **other two acyl groups**				**SDA-TAG species**		**ALA-TAG species**	
					**Mol% (0 DAI)**	**Relative amount (% of 0 DAI)**	**Mol% (0 DAI)**	**Relative amount (% of 0 DAI)**
34:0	16:0/18:0				0.5 ± 0.0	13.9 ± 0.6	0.4 ± 0.0	13.9 ± 3.6
32:0	16:0/16:0				0.6 ± 0.0	14.9 ± 1.7	0.5 ± 0.0	14.8 ± 2.8
36:0	16:0/20:0	18:0/18:0			0.5 ± 0.0	14.9 ± 2.8	0.3 ± 0.0	12.8 ± 1.8
36:1	16:0/20:1	18:0/18:1			4.2 ± 0.1	17.6 ± 2.9	4.6 ± 0.0	17.2 ± 2.5
38:1	16:0/22:1	18:0/20:1	18:1/20:0		2.1 ± 0.1	17.7 ± 1.7	1.0 ± 0.0	16.5 ± 2.6
38:4	18:2/20:2	18:3/20:1	**18:4/20:0**		7.0 ± 0.2	18.4 ± 2.4	9.7 ± 0.1	16.6 ± 2.1
34:1	16:0/18:1				4.0 ± 0.1	18.9 ± 1.8	4.3 ± 0.1	19.0 ± 3.2
36:2	16:0/20:2	18:0/18:2	18:1/18:1		7.7 ± 0.0	19.1 ± 2.3	7.9 ± 0.3	19.3 ± 2.6
38:3	18:1/20:2	18:2/20:1	18:3/20:0		10.6 ± 0.2	19.1 ± 2.2	11.7 ± 0.1	18.7 ± 2.4
38:2	18:0/20:2	18:1/20:1	18:2/20:0		8.5 ± 0.2	19.6 ± 2.1	8.1 ± 0.2	19.8 ± 2.5
38:5	18:3/20:2	**18:4/20:1**			2.9 ± 0.1	20.6 ± 2.4	2.8 ± 0.1	19.2 ± 2.4
34:2	16:0/18:2				6.2 ± 0.0	20.6 ± 2.3	6.8 ± 0.0	19.2 ± 2.0
36:3	18:0/18:3	18:1/18:2			9.4 ± 0.1	20.7 ± 2.2	10.9 ± 0.2	18.7 ± 2.1
40:2	18:1/22:1	18:2/22:0	20:0/20:2	20:1/20:1	1.6 ± 0.1	20.8 ± 3.0	0.9 ± 0.0	18.1 ± 2.7
34:3	16:0/18:3				3.6 ± 0.0	21.8 ± 2.9	4.3 ± 0.1	19.0 ± 2.7
34:4	**16:0/18:4**				1.6 ± 0.0	23.0 ± 3.4	1.4 ± 0.0	22.3 ± 1.9
36:8	**18:4/18:4**				1.1 ± 0.0	23.1 ± 3.3	0.7 ± 0.0	23.3 ± 2.9
36:7	**18:3/18:4**				3.9 ± 0.1	24.2 ± 2.6	2.9 ± 0.1	23.2 ± 2.4
36:4	**18:0/18:4**	18:1/18:3	18:2/18:2		10.2 ± 0.1	24.2 ± 1.9	10.4 ± 0.2	22.2 ± 2.4
36:6	**18:2/18:4**	18:3/18:3			6.7 ± 0.2	26.2 ± 2.6	4.5 ± 0.2	27.3 ± 2.3
36:5	**18:1/18:4**	18:2/18:3			7.2 ± 0.1	27.1 ± 2.7	6.1 ± 0.0	28.7 ± 3.2

* Sum of carbon and double bond numbers in other two acyls of SDA-TAG or ALA-TAG. The list is in order of low to high amounts of remaining SDA-containing TAG species at 4 DAI compared to 0 DAI. Species containing minor FAs 14:0, 16:1, 20:3 (0.6%), 20:4 (0.1%) were not shown. Potential combinations with at least one SDA (18:4) in other two acyls are shown in bold.

**Figure 6:**
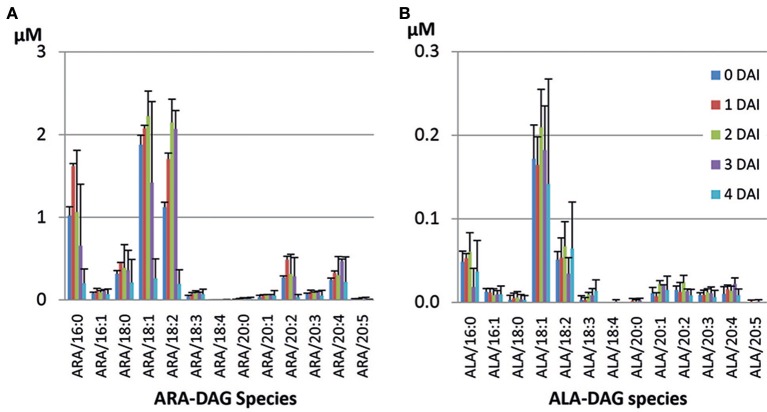
**Accumulated DAG species during seedling development in the ARA-line**. **(A)** ARA-containing DAG species, **(B)** ALA-containing DAG species.

The ARA-line was generated in MC49 background with substantially lower ALA, rather than Columbia. Comparison of TAG species within this line also showed a similar trend with slower mobilization rates of TAG species containing more PUFA. ARA-containing TAG (ARA/36:4) contained the dominant fatty acids in the seed, namely LA, OA, and ARA. This constituted the highest proportion (19.1%) among all ARA-containing TAG species (Table 4), followed by ARA/36:2 (14%). ARA-containing TAG and ALA-containing TAG species were mobilized at different levels from 0 to 4 DAI period (data not shown). At 4 DAI, most mobilized species in the seedlings were mono-ARA TAGs, which contained saturates, while di-ARA TAG species mobilized at lower proportions (Table 4). The most unused TAG at 4 DAI was tri-ARA TAG. Also, among mono- or di-ARA TAG, PUFA containing species were mobilized less compared to the species that contained saturates or MUFA. Some accumulation of di-ARA DAG was observed at 3 DAI and there was a decrease at the level at 4 DAI (Figure 6A). However, higher accumulation of overall DAG species was observed in the seedlings of ARA-line, compared to SDA-line. There were low levels of ALA-containing TAG species in the ARA-line since there was only 0.5% of ALA in seed TAG (Figure 6B). ALA/36:2 (18.9%), ALA/34:1 (12.3%), and ALA/36:3 (11.2%) were major ALA-containing TAG species, which potentially contained 16:0/20:2, 18:0/18:2, or 18:1/18:1 in 36:2, 16:0/18:1 in 34:1, and 18:1/18:2 as a major species in 36:3 (data not shown). The levels of saturates and MUFA-containing ALA-DAG species decreased at 4 DAI, while PUFA-DAG species showed accumulation (Figure 6B). The variation from triplicate ARA-line samples were relative higher than other lines (Figure 6), possibly due to the presence of more ungerminated seeds, especially at 3 or 4 DAI.

**Table 4:** **Mobilization of ARA containing TAG species on 4 DAI during seedling development in ARA-line of Arabidopsis**.

^*^Species	**Other two acyl groups**			**ARA-TAG species**	
				**Mol% (0 DAI)**	**Relative amount (% of 0 DAI)**
36:0	18:0/18:0			0.7 ± 0.0	29.3 ± 3.8
34:0	16:0/18:0			1.6 ± 0.0	30.1 ± 2.7
36:4	**16:0/20:4**	18:1/18:3	18:2/18:2	19.1 ± 0.4	32.1 ± 2.8
32:0	16:0/16:0			1.6 ± 0.0	33.5 ± 4.2
36:1	16:0/20:1	18:0/18:1		4.6 ± 0.1	33.8 ± 4.6
38:1	18:0/20:1			0.8 ± 0.0	35.0 ± 3.0
38:2	18:0/20:2	18:1/20:1		2.3 ± 0.0	35.3 ± 3.3
34:1	16:0/18:1			7.5 ± 0.1	36.0 ± 2.7
38:4	**18:0/20:4**	18:1/20:3	18:2/20:2	5.4 ± 0.1	36.8 ± 3.7
36:2	16:0/20:2	18:0/18:2	18:1/18:1	14.1 ± 0.2	39.6 ± 3.6
40:2	20:1/20:1			0.1 ± 0.0	41.7 ± 8.3
36:3	16:0/20:3	18:0/18:3	18:1/18:2	12.1 ± 0.3	41.8 ± 2.6
34:2	16:0/18:2			7.9 ± 0.1	43.0 ± 4.4
40:3	20:1/20:2			0.1 ± 0.0	43.8 ± 5.3
38:5	18:0/20:5	**18:1/20:4**	18:2/20:3	7.4 ± 0.1	44.2 ± 0.0
38:3	18:0/20:3	18:1/20:2	18:2/20:1	4.2 ± 0.1	45.1 ± 3.1
34:3	16:0/18:3			0.9 ± 0.0	45.9 ± 4.5
40:4	20:1/20:3	20:2/20:2		0.2 ± 0.0	51.0 ± 3.7
40:5	**20:1/20:4**	20:2/20:3		0.3 ± 0.0	52.1 ± 2.1
38:6	18:1/20:5	**18:2/20:4**	18:3/20:3	5.5 ± 0.1	53.3 ± 5.5
36:5	16:0/20:5	18:2/18:3		0.9 ± 0.0	55.2 ± 2.1
40:6	20:1/20:5	**20:2/20:4**	20:3/20:3	0.7 ± 0.0	60.4 ± 4.9
40:7	20:2/20:5	**20:3/20:4**		0.3 ± 0.0	61.8 ± 6.2
36:6	18:3/18:3			0.1 ± 0.0	63.0 ± 10.1
38:7	18:2/20:5	**18:3/20:4**		0.4 ± 0.0	66.5 ± 3.4
40:9	**20:4/20:5**			0.1 ± 0.0	70.7 ± 9.0
40:8	20:3/20:5	**20:4/20:4**		0.7 ± 0.0	71.8 ± 4.6

* Sum of carbon and double bond numbers in other two acyls of ARA-TAG. The list is in order of low to high amounts of remaining ARA-containing TAG species at 4 DAI compared to 0 DAI. Species containing minor FAs 14:0, 16:1, 20:0 (0.7%), 22:4(0.4%), 22:3 (0.1%), EPA (0.5%) were not shown. Potential combinations with at least one ARA (20:4) in other two acyls are shown in bold.

**Figure 7:**
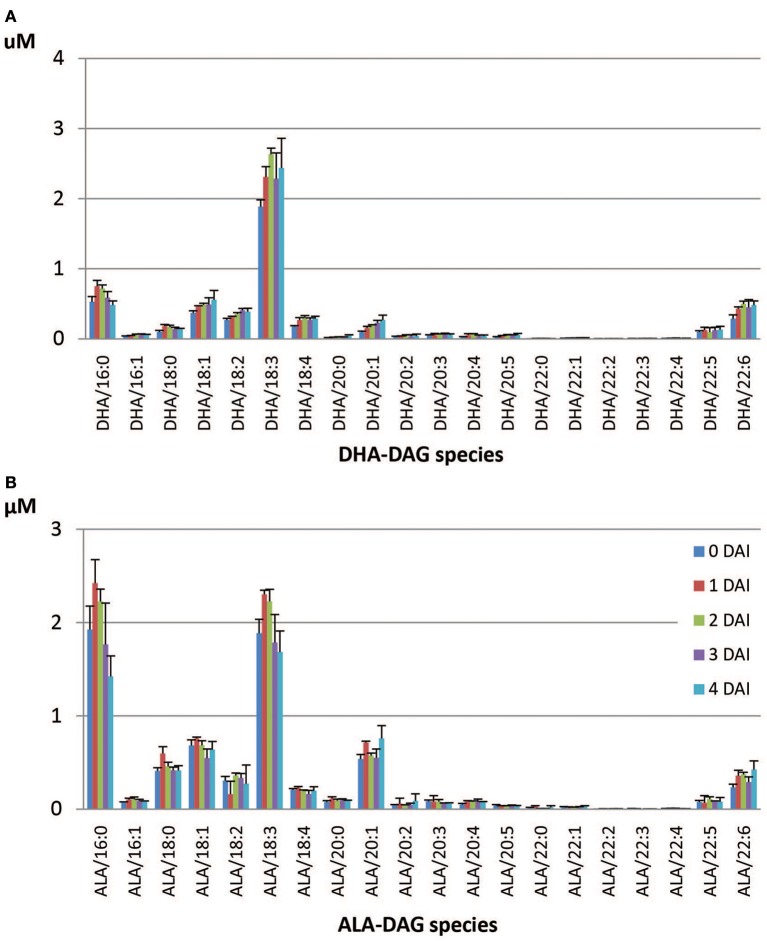
**Accumulated DAG species during seedling development in the DHA-line**. **(A)** DHA-containing DAG species, **(B)** ALA-containing DAG species.

The dominant DHA-containing TAG species in the DHA-line were DHA/36:6 (23.0%), putatively containing 18:3/18:3 and 18:2/18:4, followed by DHA/34:3 (12.5%), which putatively containing 16:0/18:3 (Table 5). All the DHA-containing TAG species were mobilized at lower levels in the DHA-line compared to SDA- or ARA-containing TAGs in the SDA-line or ARA-line at 4 DAI. Tri-DHA TAG constituted a very low proportion (0.3%) of total DHA-containing TAG species and interestingly, it was one of the least mobilized species in the seedling at 4 DAI (Table 5). Di-DHA and mono-DHA TAG species, which contained long chain PUFA were the less mobilized species. The least mobilized TAG consisted of multiple PUFA. All the DHA-containing DAG species showed accumulation during seedling growth, except for DHA/16:0 (Figure 7A). Total DAG level was higher in the DHA-line, compared to SDA- and ARA-lines. ALA/38:4 was the dominant ALA-species (15.7%) in the DHA-line, followed by ALA/34:3 (12.5%), both mono-ALA species (data not shown). Species containing two DHA (44:12) or DHA/DPA were among the least mobilized species. ALA-DAG species were not significantly changed. Some accumulation of ALA/22:6 DAG species (Figure 7B) was observed.

**Table 5:** **Mobilization of DHA containing TAG species on 4 DAI during seedling developments in DHA-line of Arabidopsis**.

^*^Species	**Other two acyl groups**					**DHA-TAG species**	
						**Mol% (0 DAI)**	**Relative amount (% of 0 DAI)**
38:9	18:4/20:5					0.2 ± 0.0	42.5 ± 7.1
36:8	18:4/18:4					0.5 ± 0.0	43.2 ± 8.5
32:0	16:0/16:0					0.5 ± 0.0	44.9 ± 5.5
36:7	18:3/18:4					5.7 ± 0.1	46.1 ± 7.3
36:5	16:0/20:5	18:1/18:4	18:2/18:3			9.5 ± 0.1	46.5 ± 8.0
34:1	16:0/18:1					2.2 ± 0.0	47.2 ± 7.5
36:0	16:0/20:0	18:0/18:0				0.1 ± 0.0	47.2 ± 1.5
36:2	18:0/18:2	18:1/18:1				4.4 ± 0.0	47.6 ± 8.2
34:4	16:0/18:4					3.0 ± 0.0	47.8 ± 6.9
34:3	16:0/18:3					12.9 ± 0.0	50.1 ± 7.4
34:0	16:0/18:0					0.2 ± 0.0	50.4 ± 6.6
38:2	18:1/20:1	18:2/20:0				1.7 ± 0.0	50.9 ± 3.0
40:10	**18:4/22:6**	20:5/20:5				0.7 ± 0.0	51.3 ± 10.3
36:6	18:2/18:4	18:3/18:3				23.0 ± 0.3	51.6 ± 8.7
34:2	16:0/18:2					1.9 ± 0.0	52.2 ± 8.1
36:1	16:0/20:1	18:0/18:1				1.4 ± 0.0	52.5 ± 7.5
38:6	**16:0/22:6**	18:1/20:5	18:2/20:4	18:3/20:3		1.8 ± 0.1	53.7 ± 7.2
36:4	16:0/20:4	18:0/18:4	18:1/18:3	18:2/18:2		7.9 ± 0.1	53.8 ± 7.3
38:8	18:3/20:5	18:4/20:4				1.0 ± 0.0	54.5 ± 5.9
38:5	16:0/22:5	18:0/20:5	18:1/20:4	18:2/20:3	18:4/20:1	2.0 ± 0.0	55.8 ± 8.2
38:1	18:0/20:1	18:1/20:0				0.3 ± 0.0	56.1 ± 6.6
36:3	16:0/20:3	18:0/18:3	18:1/18:2			5.4 ± 0.1	56.2 ± 7.0
38:3	18:0/20:3	18:2/20:1	18:3/20:0			2.0 ± 0.1	57.1 ± 7.3
40:9	**18:3/22:6**	18:4/22:5	20:4/20:5			2.9 ± 0.0	59.1 ± 8.5
40:2	20:1/20:1					0.2 ± 0.0	59.5 ± 5.1
40:6	**18:0/22:6**	18:1/22:5	20:1/20:5	20:3/20:3		0.2 ± 0.0	60.3 ± 14.0
40:4	20:0/20:4	20:1/20:3				0.2 ± 0.0	60.6 ± 6.5
38:4	18:0/20:4	18:1/20:3	18:3/20:1	18:4/20:0		4.4 ± 0.0	61.3 ± 7.0
38:7	18:2/20:5	18:3/20:4	18:4/20:3			1.0 ± 0.0	62.7 ± 9.7
40:7	**18:1/22:6**	18:2/22:5	20:3/20:4			0.5 ± 0.1	64.2 ± 2.5
44:12	**22:6/22:6**					0.3 ± 0.0	64.8 ± 10.6
42:9	**20:3/22:6**	20:4/22:5				0.1 ± 0.0	65.8 ± 6.8
40:3	20:0/20:3					0.2 ± 0.0	66.2 ± 8.4
42:7	**20:1/22:6**					0.3 ± 0.0	69.7 ± 11.7
42:11	**20:5/22:6**					0.1 ± 0.0	70.5 ± 14.7
42:10	**20:4/22:6**	20:5/22:5				0.1 ± 0.0	74.7 ± 10.8
40:5	18:0/22:5	20:0/20:5	20:1/20:4			0.2 ± 0.0	74.7 ± 7.4
42:8	20:3/22:5					0.1 ± 0.0	77.4 ± 16.1
42:6	**20:0/22:6**	20:1/22:5				0.1 ± 0.0	78.9 ± 12.0
40:8	**18:2/22:6**	18:3/22:5	20:3/20:5	20:4/20:4		0.8 ± 0.4	94.0 ± 15.3

* Sum of carbon and double bond numbers in other two acyls of DHA-TAG. The list is in order of low to high amounts of remaining DHA-containing TAG species at 4 DAI compared to 0 DAI. Species containing minor FAs 14:0, 16:1, 22:1, (0.5%), 20:2 (0.6%), 22:3 (0.1%) were not shown. Potential combinations with at least one DHA (22:6) in other two acyls are shown in bold.

### Mobilization of transgenic PUFA to membrane lipids

During the early seedling development, mobilization of the TAG in both Columbia and transgenic lines was associated with a significant increase in chloroplastic galactolipids, MGDG, DGDG, and extra-chloroplastic phospholipids, PC, PE (Table 6). However, transgenic seedlings showed overall lower amounts of total membrane lipids than Columbia. As much as 328 μg MGDG was observed in the 4 DAI seedlings, developed from 100 mg seed in Columbia, while SDA, ARA, and DHA lines contained only 76, 144, and 164 μg MGDG, respectively. DGDG, PC, and PE were also elevated in 4 DAI seedlings of Columbia compared to transgenic seedlings.

**Table 6:** **Fatty acid profiles of major membrane lipids during seedling development**.

**Class**	**Line**	**DAI**	**16:3**	**C18:2**	**C18:3n3**	**C18:3n6**	**18:4n3**	**C20:3n6**	**C20:4n6**	**20:4n3**	**20:5n3**	**22:5n3**	**C22:6n3**	**Total**
MGDG	Col	0	0.0	4.7	2.0	0.0	0.0	0.0	0.0	0.0	0.0	0.0	0.0	9.6
		4	43.6	12.5	256.9	0.0	0.0	0.0	0.0	0.0	0.0	0.0	0.0	328.4
	SDA-line	0	0.0	2.2	0.9	**0.5**	**0.4**	0.0	0.0	0.0	0.0	0.0	0.0	7.7
		4	6.5	7.8	39.5	**2.8**	**9.2**	0.0	0.0	0.0	0.0	0.0	0.0	76.4
	ARA-line	0	0.0	2.3	0.0	0.0	0.0	**0.0**	**1.4**	0.0	**0.0**	0.0	0.0	7.7
		4	22.1	10.8	87.1	0.0	0.0	**0.4**	**11.4**	0.0	**1.6**	0.0	0.0	145.0
	DHA-line	0	0.5	1.1	4.5	**0.0**	**1.2**	0.0	0.0	**0.0**	**0.0**	**0.0**	**1.1**	19.1
		4	26.5	3.1	131.7	**1.2**	**5.1**	0.0	0.0	**1.3**	**1.0**	**1.1**	**2.1**	192.3
DGDG	Col	0	0.0	2.4	1.7	0.0	0.0	0.0	0.0	0.0	0.0	0.0	0.0	8.9
		4	1.2	6.0	139.2	0.0	0.0	0.0	0.0	0.0	0.0	0.0	0.0	173.4
	SDA-line	0	0.0	1.7	1.2	**0.4**	**0.0**	0.0	0.0	0.0	0.0	0.0	0.0	7.7
		4	0.3	1.4	19.9	**0.9**	**2.5**	0.0	0.0	0.0	0.0	0.0	0.0	32.9
	ARA-line	0	0.0	0.5	0.5	0.0	0.0	**0.0**	**0.0**	0.0	**0.0**	0.0	0.0	3.2
		4	0.8	2.4	52.6	0.0	0.0	**0.2**	**1.6**	0.0	**0.3**	0.0	0.0	72.6
	DHA-line	0	0.0	0.7	2.0	**0.0**	**0.0**	0.0	0.0	**0.0**	**0.0**	**0.0**	**0.0**	8.6
		4	0.7	1.1	57.8	**0.4**	**1.3**	0.0	0.0	**0.4**	**0.3**	**0.1**	**0.3**	77.1
PC	Col	0	1.5	65.8	20.6	0.0	0.0	0.0	0.0	0.0	0.0	0.0	0.0	153.0
		4	0.0	179.7	88.4	0.0	0.0	0.0	0.0	0.0	0.0	0.0	0.0	374.9
	SDA-line	0	0.0	15.8	3.7	**4.3**	**3.0**	0.0	0.0	0.0	0.0	0.0	0.0	49.2
		4	0.0	105.5	60.4	**16.2**	**21.1**	0.0	0.0	0.0	0.0	0.0	0.0	294.2
	ARA-line	0	0.4	24.5	1.4	0.0	0.0	**2.1**	**18.1**	0.0	**0.8**	0.0	0.0	110.3
		4	0.0	113.9	10.5	0.0	0.0	**2.2**	**46.2**	0.0	**1.3**	0.0	0.0	245.1
	DHA-line	0	0.0	3.1	24.2	**0.0**	**3.1**	0.0	0.0	**0.7**	**0.7**	**3.6**	**5.9**	61.8
		4	0.0	37.3	112.1	**1.5**	**7.6**	0.0	0.0	**3.1**	**2.0**	**10.7**	**24.6**	292.6
PE	Col	0	0.7	32.1	7.5	0.0	0.0	0.0	0.0	0.0	0.0	0.0	0.0	64.1
		4	0.3	122.3	40.3	0.0	0.0	0.0	0.0	0.0	0.0	0.0	0.0	262.7
	SDA-line	0	0.0	13.2	2.5	**3.6**	**1.7**	0.0	0.0	0.0	0.0	0.0	0.0	37.3
		4	0.0	59.8	23.1	**8.7**	**6.5**	0.0	0.0	0.0	0.0	0.0	0.0	162.7
	ARA-line	0	0.0	13.8	0.9	0.0	0.0	**1.1**	**12.4**	0.0	**0.0**	0.0	0.0	58.3
		4	0.0	84.0	3.0	0.0	0.0	**1.4**	**31.5**	0.0	**0.7**	0.0	0.0	182.7
	DHA-line	0	0.4	2.5	20.4	**0.0**	**3.1**	0.0	0.0	**0.5**	**1.0**	**3.0**	**10.3**	64.2
		4	0.2	30.0	62.2	**1.1**	**3.4**	0.0	0.0	**1.5**	**1.6**	**5.3**	**19.9**	197.9

Amount in μg/100 mg seed. Only major endogenous (16:3, 18:2, 18:3n3) and engineered fatty acids (in Bold) were shown.

Significant amounts of the engineered fatty acids in TAG were mobilized to build up the membrane lipids, as a big surge in the contents of engineered fatty acids in both plastidic and extra-plastidic membrane lipids were observed (Table 6). Up to 9.19 μg and 2.51 μg SDA were mobilized into MGDG and DGDG, respectively, in SDA-line. ARA was mobilized 11.42 and 1.55 μg into MGDG and DGDG in ARA-line, while only 2.39 and 0.28 μg DHA were mobilized into MGDG and DGDG in DHA-line. LC-MS analysis of MGDG and DGDG (Figure S2) confirmed the overall lower amounts of membrane lipids in transgenic seedlings when compared with Columbia, as well as the engineered fatty acids appearing in MGDG or DGDG. SDA appeared in both MGDG (mainly 34:7 and 36:7) and DGDG (mainly 36:7) while ARA appeared mainly in MGDG (mainly 38:7 and small amount of 36:7). DHA only accumulated very low level in MGDG and DGDG (mainly 40:9). Unlike the endogenous major membrane lipid fatty acid components which could be *de novo* synthesized in addition to reusing from TAG mobilization, the engineered fatty acids in MGDG or DGDG, and the increased amount in PC or PE could only be from TAG mobilization, as the transgenes were expressed specifically in developing seeds, but not in seedlings.

## Discussion

Seed TAG mobilization is catalyzed by seed specific lipases and is vital for the early stages of seedling growth. Seed lipases have shown differential specificity for TAGs containing various fatty acids in *in vitro* studies, suggesting that lipases are more selective to the TAG species which contain fatty acids naturally present in the seed of a given plant species. TAG lipases are believed to be located in the oil body membranes where *in vivo* lipolysis occurs at an oil/water interphase. In contrast, TAG substrates are not very soluble in the aqueous phases of *in vitro* assays and can also be exposed to various other lipases from biological samples, possibly leading to difficulty in assessing activities of specific TAG lipases. Here, we have avoided these difficulties by studying TAG mobilization *in vivo* using transgenic strains producing unusual fatty acids.

Seed fatty acid profiling and oil quantification showed the presence of substantial levels of transgenic PUFA and also revealed typically lower levels of oil content when compared with control Arabidopsis seeds. TAG mobilization tended to be reduced as the chain length and desaturation of engineered fatty acids increased even within the same line (Figure 2), likely owing to the presence of PUFA in TAG. The slower TAG mobilization observed in transgenic lines, together with the lower proportions of PUFA liberation from TAG in such lines, suggested lower specificity of seed or seedling lipases to the transgenic fatty acids accumulated in TAG and at the same time indicated preferential specificities for shorter chain and saturated fatty acids. The slower seedling growth rates observed in transgenic lines, especially in the ARA-line, might be due to the presence of lower level of TAG in the seeds of this particular line and/or due to slower rate of TAG mobilization, compared to Columbia.

Lower level of utilization of both the major transgenic fatty acids in the SDA-line seed, GLA and SDA, compared to endogenous fatty acids such as ALA indicated lower specificities of seed lipase to those fatty acids. This may have resulted in the lower level of TAG mobilization during seedling development, which ultimately led to the slower rate of seedling development. This phenomenon was more pronounced in the ARA-line, where ω6 PUFA ARA was the major engineered fatty acid and significantly lower amount of ARA liberated from TAG in comparison to normal fatty acids. As a result, both seed germination and seedling development were retarded. Arabidopsis seeds with even higher levels of ARA (38%) showed slower germination (Naim et al., 2016). Similarly, transgenic PUFAs were utilized at a lesser degree compared with normal seed TAG fatty acids in the DHA-line. However, ALA and DHA were liberated at similar degrees (35 and 32%) at 3 DAI. In the DHA-line, the major DHA-TAG species were formed together with ALA and higher level of utilization of DHA could be due to its association with ALA. It is important to highlight that at 0 DAI the PUFAs in TAG were already slightly higher in proportion than in dry seeds where the TAG accounted for normally 97% of total lipid, while the profiles of short chain saturate or monounsaturated fatty acids were slightly decreased, for example, 15.7% of ARA in dry seed of ARA-line or 11.6% of DHA in dry seed of DHA-line (Table 1) were increased to 19.6 or 13.9% at 0 DAI, respectively (Figure 1). This suggested the TAG mobilization had already occurred during the imbibition, although the mobilization levels were low. During this imbibition period, slower mobilization of PUFA, especially the engineered fatty acids occurred compared to short chain saturate or monounsaturated fatty acids.

Overall, TAG species study using LC-MS showed slower utilization of di- or tri-PUFA-containing species compared to single PUFA TAG in general. Some accumulation of di-SDA, di-ARA, or di-DHA DAG was observed. Di-SDA DAG concentration had no obvious change from 0 to 4 DAI, while other SDA-containing DAG species were reduced (Figure 5). ARA/18:2 DAG and di-ARA DAG increased from 0 to 4 DAI, while some other ARA-containing DAG reduced (Figure 6). Di-DHA DAG was also increased (Figure 7). This was likely due to the preference of TAG lipase to non-engineered normal fatty acids from their respective TAG species that contained two engineered fatty acids. This also indicated the lower specificity lipase to transgenic PUFA. Production of higher levels of these fatty acids in seeds could build up more di- or tri-PUFA TAG species and might show negative implications on seedling development and establishment. The slower mobilization rate toward PUFA could also been observed in Columbia, as tri-ALA TAG was accumulated more than other TAG species at 4 DAI relative to 0 DAI (Table 2). The mobilization rates of engineered PUFA with longer chains and more double bonds were even slower.

During seedling development, *de novo* fatty acid synthesis occurs at the later stages. There is a possibility that a proportion of these newly synthesized fatty acids also contributed to new TAG synthesis so the TAG observed at later stages of seedling development might be the combined amount of residual seed TAG plus newly synthesized TAG. However, there should be no newly synthesized engineered PUFA in seedling vegetative tissues, as the transgenic pathways were expressed by seed-specific promoters. Therefore, the observed slower mobilization of engineered PUFA would solely be the effect of lower lipase preference toward these fatty acids.

Another hypothesis tested was whether the engineered PUFA released from TAG was reused by the membrane lipid synthesis. During early seedling development, mobilization of the TAG was associated with a significant increase in all lines of the membrane lipids, namely chloroplastic galactolipids, MGDG, DGDG, and extra-chloroplastic phospholipids, PC, PE. The increased MGDG 34:6 (16:3/18:3) or 36:6 (18:3/18:3) and DGDG 36:6 (18:3/18:3) could be from *de novo* synthesized FA. If the engineered FA were used, MGDG or DGDG 36:7 (18:3/SDA), 38:7 (18:3/ARA), or 40:9 (18:3/DHA) from SDA, ARA, or DHA line would be expected. The results showed some low level (albeit increasing) amounts of those lipids i.e., MGDG 36:7 in SDA line, MGDG 38:7 in ARA line, as well as trace amount of MGDG 40:9 in DHA line, which would be converted from TAG lysis products. Fatty acids coming from both *de novo* synthesis and storage TAG origins should have roles in the membrane lipid synthesis. Accumulation of engineered fatty acids in the membrane lipids indicated the mobilization of seed TAG for membrane lipid synthesis. As the PUFA pathway were engineered to express specifically in developing seeds, the engineered fatty acids in the membrane lipids during seedling development should be coming from the TAG store. Compared to Columbia, all transgenic seedlings showed lower levels of membrane lipids, especially MGDG and DGDG with significant reduction, even though there might be partially contributed by some ungerminated seeds, which were found in both Columbia and transgenic lines. However, significant increase in the amounts of engineered fatty acids were observed in all analyzed membrane lipid classes including chloroplastic membrane lipids, indicating the role of storage TAG in the membrane building during early stage of seedling development. Similar results were also observed in the green microalga *Parietochloris incisa*, where ARA (ω6 PUFA) was swiftly mobilized from storage TAG to plastidic galactolipids during the onset of logarithmic growth (Khozin-Goldberg et al., 2005). Slower mobilization of TAG and its fatty acids in the transgenic seedlings might have impacts on membrane lipid synthesis, which could turn into slower development of seedlings. In addition, the presence of engineered PUFA in membrane lipids might have effects in the normal growth of seedlings.

New groups of long chain PUFA-containing PC were observed in seedlings of SDA and ARA in a manner similar to that observed in developing Arabidopsis seeds expressing the DHA pathway (Zhou et al., 2014, and data not shown). The engineered PUFA containing PC also had a slower decrease rate during the seedling development, especially PCs containing ARA.

In summary, our work showed slower TAG mobilization rate for the TAG species containing more engineered PUFA. Higher amount of TAG with engineered PUFA might eventually slow down the seedling development process. TAG lipases with higher specificity for engineered fatty acids should be in consideration for better crop engineering. Also, the higher seed oil contents in oil crop species may affect the relative importance of PUFA being spared in such transgenic events.

## Author contributions

Conceived and designed the experiment: PS, JP, SS, XZ. Performed the experiment: PS, DC, XZ. Analyzed data: PS, DC, JP, SS, XZ. Wrote the paper PS, XZ. All authors revised the draft and approved the final manuscript.

### Conflict of interest statement

The authors declare that the research was conducted in the absence of any commercial or financial relationships that could be construed as a potential conflict of interest.

## Supplementary material

Figure S1 **Seedling development in Columbia and the transgenic Arabidopsis lines after imbibition**. DAI, day after imbibition; Col, Columbia; SDA, ARA, DHA are the transgenic lines expressing relevant pathways; Null-DHA is the null segregant from DHA-line.

Figure S2 **Biosynthesis of PUFA-containing MGDG and DGDG species during seedling development in Columbia, SDA-, ARA-, and DHA-Arabidopsis lines**. The two numbers of the species denotes total number of carbons and total number of double bonds of two fatty acids in the species. **(A)** MGDG; **(B)** DGDG.
