# Supplementary figures and images for: Reduced Triacylglycerol Mobilization during Seed Germination and Early Seedling Growth in Arabidopsis Containing Nutritionally Important Polyunsaturated Fatty Acids
Source: Front Plant Sci. 2016 Sep 26;7:1402. doi: 10.3389/fpls.2016.01402 (PMC5035741; doi:10.3389/fpls.2016.01402)

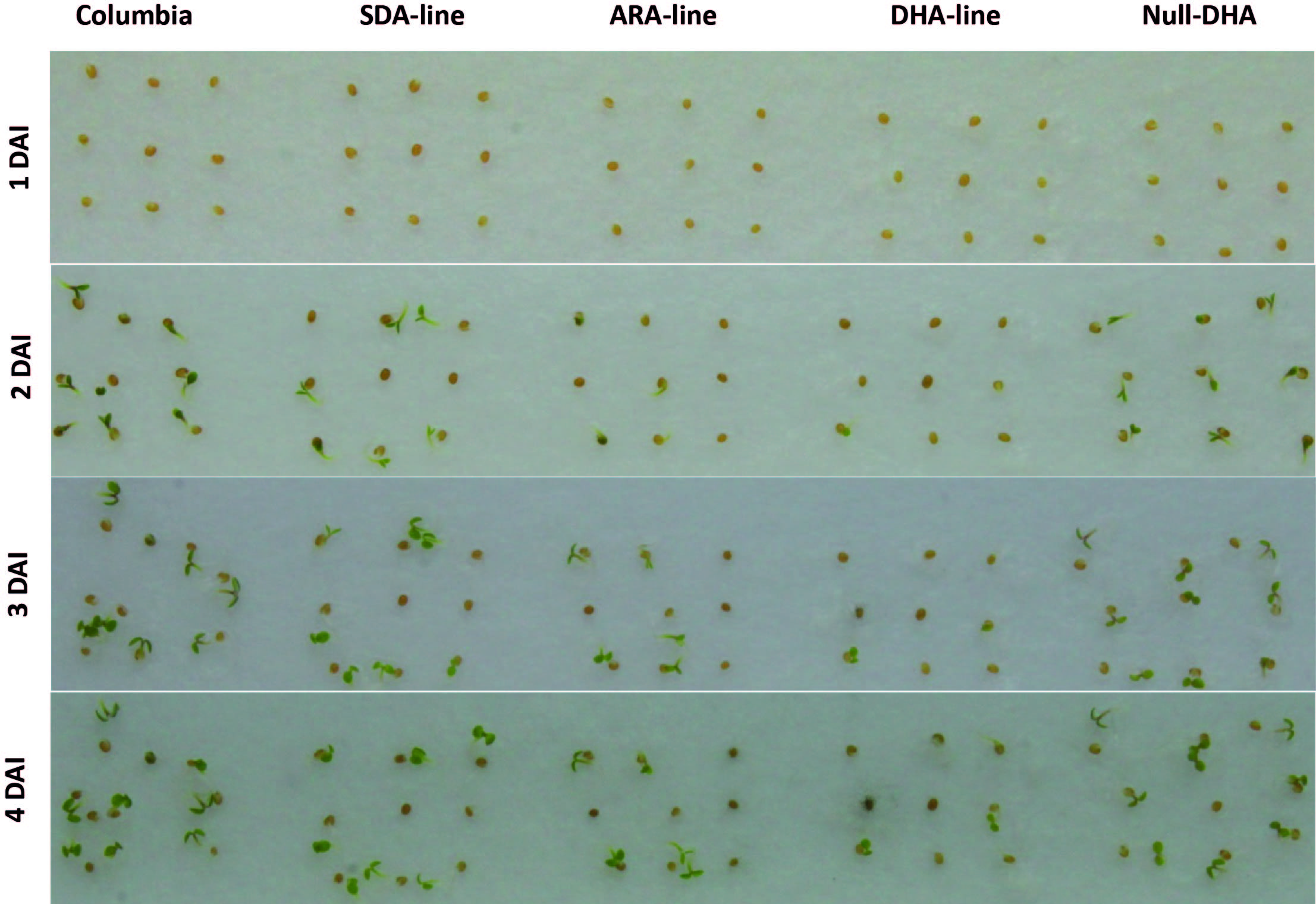

Supplement: Figure S1 — Seedling development in Columbia and the transgenic Arabidopsis lines after imbibition. DAI, day after imbibition; Col, Columbia; SDA, ARA, DHA are the transgenic lines expressing relevant pathways; Null-DHA is the null segregant from DHA-line. [file Image1.JPEG]

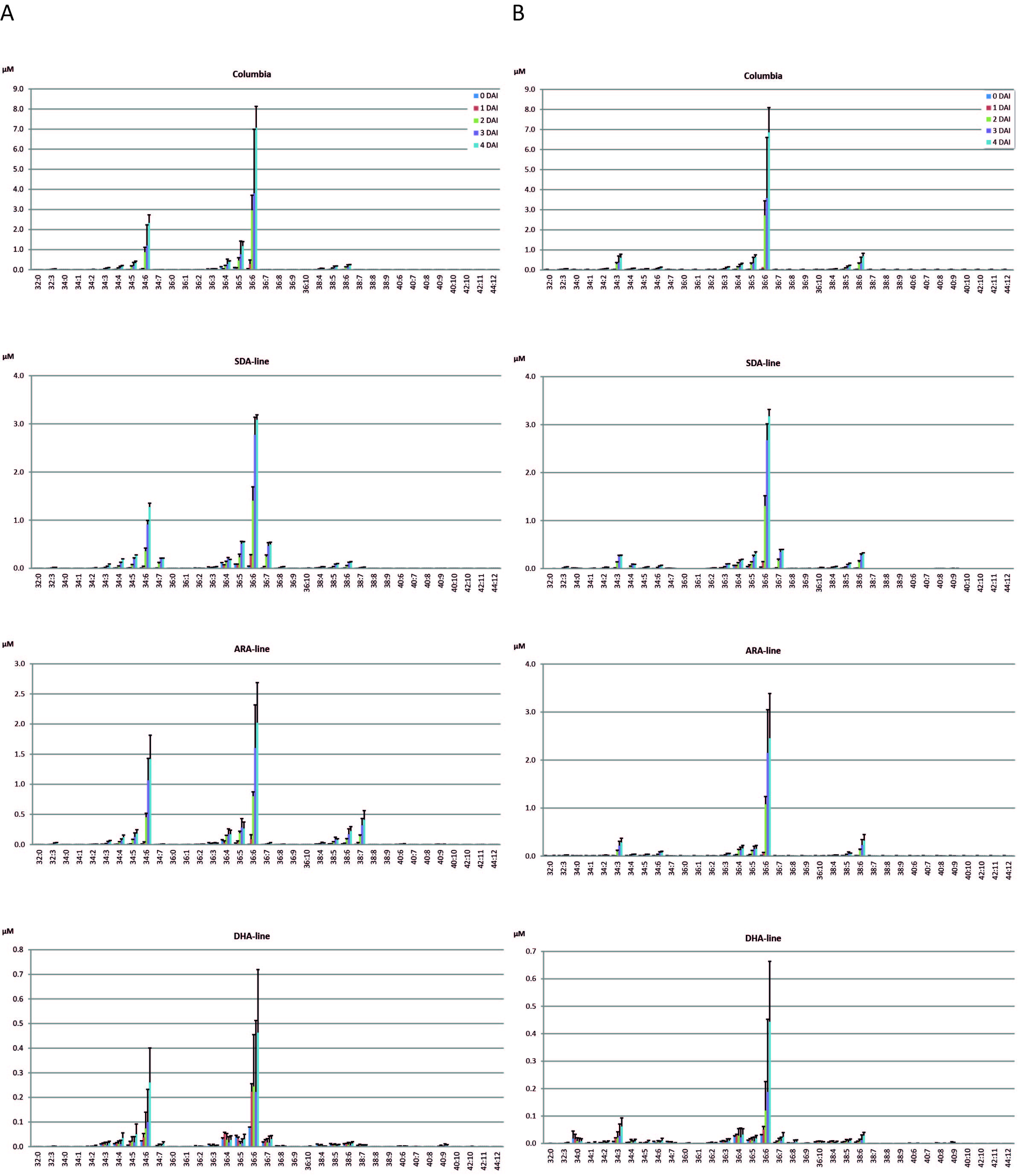

Supplement: Figure S2 — Biosynthesis of PUFA-containing MGDG and DGDG species during seedling development in Columbia, SDA-, ARA-, and DHA-Arabidopsis lines. The two numbers of the species denotes total number of carbons and total number of double bonds of two fatty acids in the species. (A) MGDG; (B) DGDG. [file Image2.JPEG]
